# The complete chloroplast genome sequence of *Pellacalyx yunnanensis*: an endangered species in China

**DOI:** 10.1080/23802359.2019.1688110

**Published:** 2019-11-12

**Authors:** Jinfeng Zhang, Yunqing Li, Xiaolong Yuan, Yi Wang

**Affiliations:** Laboratory of Forest Plant Cultivation and Utilization, Yunnan Academy of Forestry, Kunming, People’s Republic of China

**Keywords:** *Pellacalyx yunnanensis*, chloroplast, Illumina sequencing, phylogenetic analysis

## Abstract

*Pellacalyx yunnanensis* is an endangered plant species occurring in Yunnan province of China. The first complete chloroplast genome (cpDNA) sequence of *P. yunnanensis* was determined from Illumina HiSeq pair-end sequencing data in this study. The cpDNA is 163,743 bp in length, contains a large single-copy region (LSC) of 91,075 bp and a small single-copy region (SSC) of 18,668 bp, which were separated by a pair of inverted repeats (IR) regions of 27,000 bp. The genome contains 128 genes, including 83 protein-coding genes, 8 ribosomal RNA genes, and 37 transfer RNA genes. The overall GC content of the whole genome is 35.7%, and the corresponding values of the LSC, SSC, and IR regions are 33.0%, 29.6%, and 42.3%, respectively. Further phylogenomic analysis showed that *P. yunnanensis* clustered in a unique clade in family *Rhizophoraceae*.

*Pellacalyx yunnanensis* is the species of the genus *Pellacalyx* within the family Rhizophoraceae. It is terrestrial species in Rhizophoraceae family (Yang et al. 2015). *Pellacalyx yunnanensis* is endemic to Yunnan of China and distributed in Xishuangbanna of Yunnan Province (Su et al. 2005). It also is a rare and endangered plant of the family Rhizophoraceae (Qin et al. 2017). *Pellacalyx yunnanensis* is of great value to the study of tropical flora in China (Ma et al. 1988). However, there have been no genomic studies on *P. yunnanensis*.

Herein, we reported and characterized the complete *P. yunnanensis* plastid genome (MN106253). One *P. yunnanensis* individual (specimen number: 201807012) was collected from Jinghong, Yunnan Province of China (22°49′38′′ N, 101°9′27′′ E). The specimen is stored at Yunnan Academy of Forestry Herbarium, Kunming, China and the accession number is YAFH0012753. DNA was extracted from its fresh leaves using DNA Plantzol Reagent (Invitrogen, Carlsbad, CA).

Paired-end reads were sequenced by using Illumina HiSeq system (Illumina, San Diego, CA). In total, about 27.9 million high-quality clean reads were generated with adaptors trimmed. Aligning, assembly, and annotation were conducted by CLC de novo assembler (CLC Bio, Aarhus, Denmark), BLAST, GeSeq (Tillich et al. 2017), and GENEIOUS version 11.0.5 (Biomatters Ltd, Auckland, New Zealand). To confirm the phylogenetic position of *P. yunnanensis*, other six species of order *Malpighiales* from NCBI were aligned using MAFFT version 7 (Katoh and Standley 2013). The Auto algorithm in the MAFFT alignment software was used to align the eight complete genome sequences and the G-INS-i algorithm was used to align the partial complex sequecnces and maximum likelihood (ML) bootstrap analysis was conducted using RAxML (Stamatakis 2006); bootstrap probability values were calculated from 1000 replicates. *Parnassia trinervis* (MH544205) and *Euonymus schensianus* (KY511610) were served as the out-group.

The complete *P. yunnanensis* plastid genome is a circular DNA molecule with the length of 163,743 bp, contains a large single-copy region (LSC) of 91,075 bp and a small single-copy region (SSC) of 18,668 bp, which were separated by a pair of inverted repeats (IR) regions of 27,000 bp. The overall GC content of the whole genome is 35.7%, and the corresponding values of the LSC, SSC, and IR regions are 33.0%, 29.6%, and 42.3%, respectively. The plastid genome contained 128 genes, including 83 protein-coding genes, 8 ribosomal RNA genes, and 37 transfer RNA genes. Phylogenetic analysis showed that *P. yunnanensis* clustered with other three species of family Rhizophoraceae together and clustered in a unique clade in family Rhizophoraceae (Figure 1). The determination of the complete plastid genome sequences provided new molecular data to illuminate the Malpighiales evolution.

**Figure 1.**
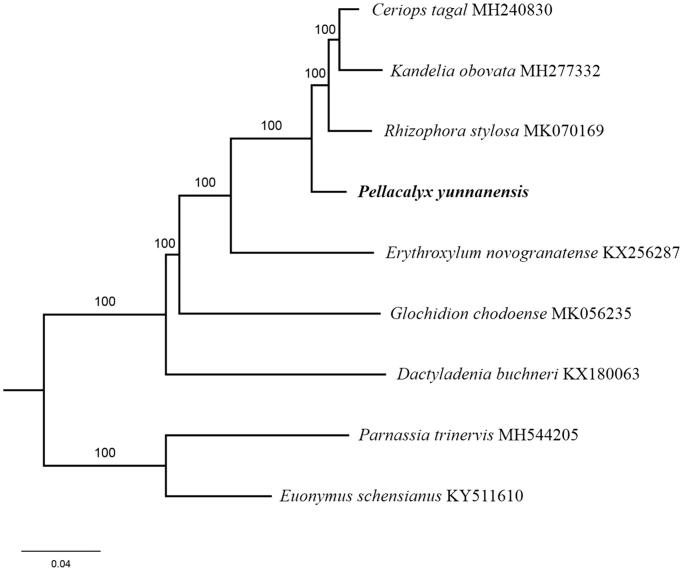
The maximum-likelihood tree based on the seven chloroplast genomes of Malpighiales. The bootstrap value based on 1000 replicates is shown on each node.
